# Neuroprotective potential of isothiocyanates in an in vitro model of neuroinflammation

**DOI:** 10.1007/s10787-020-00772-w

**Published:** 2020-11-16

**Authors:** Tiziana Latronico, Marilena Larocca, Serafina Milella, Anna Fasano, Rocco Rossano, Grazia Maria Liuzzi

**Affiliations:** 1 Department of Biosciences, Biotechnologies and Biopharmaceutics, University of Bari “Aldo Moro”, Bari, Italy; 2 Department of Sciences, University of Basilicata, Potenza, Italy

**Keywords:** Antioxidant, Anti-inflammatory, Isothiocyanate, Matrix metalloproteinases, Neurodegenerative diseases

## Abstract

Isothiocyanates (ITCs), present as glucosinolate precursors in cruciferous vegetables, have shown anti-inflammatory, antioxidant and anticarcinogenic activities. Here, we compared the effects of three different ITCs on ROS production and on the expression of matrix metalloproteinase (MMP)-2 and -9, which represent important pathogenetic factors of various neurological diseases. Primary cultures of rat astrocytes were activated by LPS and simultaneously treated with different doses of Allyl isothiocyanate (AITC), 2-Phenethyl isothiocyanate (PEITC) and 2-Sulforaphane (SFN). Results showed that SFN and PEITC were able to counteract ROS production induced by H_2_O_2_. The zymographic analysis of cell culture supernatants evidenced that PEITC and SFN were the most effective inhibitors of MMP-9, whereas, only SFN significantly inhibited MMP-2 activity. PCR analysis showed that all the ITCs used significantly inhibited both MMP-2 and MMP-9 expression. The investigation on the mitogen-activated protein kinase (MAPK) signaling pathway demonstrated that ITCs modulate MMP transcription by inhibition of extracellular-regulated protein kinase (ERK) activity. Results of this study suggest that ITCs could be promising nutraceutical agents for the prevention and complementary treatment of neurological diseases associated with MMP involvement.

## Introduction

Neuroinflammation is a complex response to brain injury involving a cascade of biochemical events leading to the activation of central nervous system (CNS) resident cells. The aberrant activation of astrocytes compromises their neuroprotective role leading to release of inflammatory mediators, such as reactive oxygen species (ROS), nitric oxide (NO), cytokines, chemokines and matrix metalloproteinases (MMPs).

MMPs are a large family of neutral, Zn^2+^-dependent endopeptidases that have as major targets the components of the extracellular matrix (ECM) such as fibronectin, collagen, elastin and laminin (Latronico and Liuzzi 2017). In the CNS, MMPs are involved in various physiological responses, such as morphogenesis, turnover and remodelling of ECM, embryogenesis and wound repair; however, when MMPs escape regulatory mechanisms they become harmful (Rosenberg 2009; Agrawal et al. 2008).

In this respect, it is known that alterations in MMP expression and activity are key pathogenetic events in several neurological disorders (Latronico and Liuzzi 2017; Mastroianni and Liuzzi 2007; Brkic et al. 2015). Experimental evidence has suggested that among MMPs, gelatinases A (MMP-2) and B (MMP-9) are particularly involved in neuroinflammation since their upregulation may compromise the integrity of blood brain barrier (BBB). This leads to increased recruitment and infiltration of immune peripheral blood cells into the brain parenchyma perpetuating the inflammatory process that is cause of progressive neuronal loss and impaired neuronal function (Rivest 2009). Several in vitro and in vivo studies have shown ROS production is one of the factors involved in the up-regulation of MMP-9 expression in brain cells via mitogen activated protein kinase (MAPK) signaling pathway dependent mechanisms (Hsieh and Yang 2013).

Based on this evidence, it is clear that downregulation of neurotoxic factors, resulting in reduced neuroinflammatory responses, may represent an effective therapeutic strategy to prevent the onset or alleviate the progression of brain diseases.

In recent years, the biomedical interest in the neurological field is increasingly focused on research of natural compounds able to modulate the processes associated with neuroinflammatory responses. In this respect, several studies have shown that phytochemical compounds, such as polyphenols, isothiocyanates (ITCs) and others, have neuroprotective potential which also derives from their ability to inhibit the expression of MMPs and to counteract ROS production (Dinkova-Kostova and Kostov 2012; Liuzzi et al. 2007, 2011).

Isothiocyanates are metabolites produced by several plants belonging to the Brassicaceae families as a system of defense against pathogen attack (Bones and Rossiter 2006). They originate from the enzymatic degradation of glucosinolates (GSL) by the enzyme myrosinase. ITCs are present in commonly consumed cruciferous vegetables such as broccoli, Brussels sprouts, cabbage, turnip, horseradish, kohlrabi, mustard, radish, watercress and kale. Their amounts in food are very variable and dependent on food processing and preparation.

Recently it has been showed that ITCs possess a variety of therapeutic effects, including antioxidant, antibacterial, anti-inflammatory and chemopreventive action (Dufour et al. 2015; Marzocco et al. 2015).

The protective activity of ITCs is carried out through modulation of different signaling pathways involved in detoxification, inflammation, apoptosis and cell cycle regulation. Several evidences indicate that the anti-carcinogenic and anti-inflammatory properties of ITCs could be mainly ascribed to their ability to activate the nuclear factor erythroid 2-related factor 2 (Nrf2) pathway. Nrf2 is a redox-sensitve transcriptional factor that in presence of electrophilic agents and oxidative stress conditions translocates into the nucleus where it binds the antioxidant response elements (ARE), inducing the expression of cytoprotective genes involved in detoxification and in the modulation of oxidative conditions and inflammatory processes (Heiss et al 2001). In this respect, it has been reported that ITCs are able to increase the antioxidant capacity of human cells by inducing the expression of phase II detoxifying enzymes and by the up-regulation of GSH production (Wagner et al 2010). Furthermore, it has been reported the existence of a cross-talk between Nrf2 and the nuclear factor (NF)-κB pathway that results in the modulation of inflammation (Sturm and Wagner 2017; Lee and Johnson 2004). In this respect, various experimental in vitro and in vivo models of neuroinflammation have demonstrated that ITCs are able to significantly decrease NF-κB translocation with the consequent inhibition of proinflammatory cytokines and MMPs (Lee et al. 2015; Subedi et al. 2017).

In this paper we investigated the effect of three ITCs i.e. Allyl isothiocyanate (AITC), 2-Phenethyl isothiocyanate (PEITC) and 2-Sulforaphane (SFN) on the release of MMP-2 and -9 as well as on ROS production by LPS-activated astrocytes. Our results evidenced that PEITC, SFN and AITC are able to inhibit in vitro the expression of MMP-9 and MMP-2 in LPS-activated astrocytes and to counteract ROS production induced by H_2_O_2_. In addition, we demonstrated that ITCs modulate the overexpression of MMPs with mechanisms related to inhibition of the extracellular-regulated protein kinase (ERK) activity. These results suggest that a diet rich in Cruciferous vegetables may have potential benefits for the prevention and complementary treatment of neurological diseases.

## Materials and methods

### Chemicals and reagents

Dulbecco's modified Eagle's medium (DMEM), fetal bovine serum (FBS), penicillin and streptomycin were provided by GIBCO (Paisley, Scotland). Gelatin, DNase 1, poly-L-lysine (PLL), trypsin, lipopolysaccharide (LPS), Trypan Blue and 3-(4,5-dimethylthiazol-2-yl)-2.5-diphenyltetrazolium bromide (MTT), Allyl isothiocyanate (AITC) [cod. 377,430, purity 95% (HPLC)], 2-Phenethyl isothiocyanate (PEITC) [cod. 253,731, purity 99%)], 2-Sulforaphane (SFN) [cod. S6317, purity ≥ 95% (HPLC)] were provided from Sigma (St. Louis, MO, U.S.A.). 2′,7′-dichlorofluorescein diacetate (DCFH-DA) was purchased from Calbiochem. Standard proteins and R-250 Coomassie Brilliant Blue were from Bio-Rad (Hercules, CA, USA). Anti-glial fibrillary acidic protein (GFAP) antibodies (RRID: AB_2294571) were purchased from Serotec (Oxford, UK). Antibodies against extracellular–regulated protein kinases (ERK) 1/2, and phosporylated ERK 1/2 (p-ERK 1/2) were from Santa Cruz Biotechnology (Santa Cruz, CA). Hybond-P PVDF membranes, enhanced chemiluminescence (ECL) Western Blotting Analysis System and anti-mouse-HRP secondary antibody were from GE Healthcare Life Sciences (Little Chalfont, Buckinghamshire, UK). Primer pairs specific for MMP-2, MMP-9 and 18S rRNA were from Sigma Genosys (Cambs, UK). RNeasy mini kit and QuantiTect Reverse Transcription kit were purchased from Qiagen (Valencia, CA, USA). The EconoTaq PLUS GREEN 2X Master Mix reagents for PCR was purchased from Lucigen Corporation (Middleton, WI, USA).

### Ethics statement

The experiments involving animals were carried out according to the recommendations in the NIH Guide for the Care and Use of Laboratory Animals and with approval of the Institutional Animal Care and Use Committee of University of Bari, Italy (Permit Number: 23-98-A).

### Preparation of astrocyte cultures

Astrocytes were prepared from the neocortical tissues of 1-day-old Wistar rats (RRID: RGD_737960, Harlan Laboratories srl, Udine, Italy). For the preparation of astrocytes 6 litters of 12 pups each of both sexes were used. The animals were euthanized by exposure to carbon dioxide (CO_2_) and killed by rapid decapitation. The neocortical tissues were dissected and used for purification of primary glial cell cultures following the method reported by Latronico et al. (2018). Briefly, after the dissection, rat brains were depleted of meninges and blood vessels, mechanical minced and digested with 0.25% trypsin in presence of 0.01% DNase. After centrifugation and assessment of cell viability, cells were plated in PLL-coated flasks (75 cm^2^) at a density of 1.5 × 10^7^ cells/flask in DMEM, 10% FBS, 100 IU mL^−1^ penicillin, 100 µg mL^−1^ streptomycin, and maintained at 37 °C in a 5% CO_2_. After 7 days, the flasks were shaken to remove oligodendrocytes and microglia. The purity of astrocytes, obtained by trypsinization (0.25% trypsin/0.02% EDTA), was assessed by immuno-staining for GFAP.

### Treatment of LPS-activated astrocytes with Allyl isothiocyanate, Phenethyl isothiocyanate or 2-Sulforaphane

Confluent astrocytes, seeded in 96 well-plates, were stimulated with 10 µg mL^−1^ of LPS and simultaneously treated with different concentrations of Allyl isothiocyanate (AITC), 2-Phenethyl isothiocyanate (PEITC) or 2-Sulforaphane (SFN) in serum-free DMEM. Astrocytes in serum-free DMEM and LPS-activated astrocytes represented negative and positive controls, respectively. In addition, since the stock solutions of AITC (100 µM), PEITC (67 µM) and SFN (56.4 µM) were made in DMSO, a dose–response curve to DMSO, diluted in culture medium at the same solvent concentration present in the analyzed samples, was run to estimate compound toxicity. After 20 h of incubation at 37 °C, 5% CO_2_ the supernatants were collected and stored at -20 °C until use whereas the cells were subjected to MTT assay to assess cell viability.

### MTT cell viability assay

Cytotoxicity of AITC, PEITC and SFN on astrocytes was detected using the MTT [3-(4,5-dimethylthiazol-2-yl)-2,5-diphenyl tetrazolium bromide] assay. This assay is based on the reduction of MTT by the mitochondrial succinate dehydrogenase in viable cells, to an insoluble blue formazan product which can be spectrophotometrically measured. Briefly, after treatment for 20 h with AITC, PEITC or SFN, the culture medium was removed and cells were loaded with 0.5 mg mL^−1^ of MTT. After incubation for 2 h at 37 °C, 5% CO_2_ medium was removed and the formazan crystals in cells were solubilized with 90% ethanol. After shaking of plates for 15 min to obtain a complete solubilization of crystals, the quantity of the formazan product was determined by optical absorbance at 560 nm with a reference wavelength of 690 nm. Cell viability was expressed as percentage of control (CTRL), represented by untreated cells, which was set at 100%. For each compound, a dose–response curve of cell viability was obtained.

### Reactive oxygen species detection

Detection of reactive oxygen species (ROS) was performed by loading astrocytes with 10 μM of 2′,7′-dichlorofluorescein diacetate (DCFH-DA) in phenol red–free DMEM at 37 °C for 30 min. Then DCFH-DA was removed from wells and cells were treated for 1 h with AITC (400 μM), PEITC (10 μM) or SFN (25 μM) in phenol red–free DMEM in presence of H_2_O_2_ at the final concentration of 100 μM. Cells treated only with DCFH-DA or with H_2_O_2_ represented the negative (CTRL) and positive controls (H_2_O_2_), respectively. After incubation cells were rinsed with PBS and lysed with Tris–HCl 10 mM/NaCl 150 mM/Triton X-100 0.5%, pH 7.5, then centrifuged at 10,000×*g*, 4 °C for 10 min. Supernatants were collected and their spectrofluorimetric analysis was performed at 525 nm under excitation at 485 nm. Results were normalized to total protein content and ROS production was expressed as relative percentage of photoluminescence (PL) intensity versus positive control.

### Detection of gelatinases

Gelatinase activity in cell supernatants (SNs) was detected by zymographic analysis as reported by Latronico et al. (2013). Briefly, an amount of SN, corresponding to about 10 µg of total proteins, was solubilized with 30 µl of Laemmli sample buffer without β-mercaptoethanol. Samples were run in a 7.5% polyacrylamide gel copolymerized with 0.1% (wt/v) gelatin. After the electrophoretic run, gels were rinsed twice with 2.5% Triton X-100/10 mM CaCl_2_ in 50 mM Tris–HCl, pH 7.4 and incubated for 24 h at 37 °C in 1% Triton X-100/50 mM Tris–HCl/10 mM CaCl_2_, pH 7.4. The gels were stained with Coomassie brilliant blue R-250 and destained in methanol/acetic acid/H_2_O (4:1:5 v/v). Gelatinase activity was visualized as a clear band of digestion on a blue background of the gel and was quantified by computerized densitometric image analysis using Image LabTM Software (Bio-Rad Laboratories). Gelatinase activity was expressed as optical density (OD) × mm^2^, representing the scanning area under the curves which takes into account both brightness and width of the substrate lysis zone. Data were expressed using the following equation: % inhibition = [100 − (OD sample/OD positive control) × 100].

### Reverse transcription-polymerase chain reaction (RT-PCR)

Astrocytes, seeded in 6 well plates, were activated with LPS (10 µg mL^−1^) and simultaneously treated with ITCs at the maximum non-toxic concentration. After 20 h of incubation total RNA was extracted from astrocytes using the Qiagen RNeasy mini kit according to the manufacturer’s instructions. Complementary DNA (cDNA) was synthesized from 500 ng of RNA by using the QuantiTect Reverse Transcription kit according to manufacturer’s instructions. A total of 25 ng of reverse transcription products were used to amplify a 591 bp fragment using specific primers (sense 5′-GTC ACT CCG CTG CGC TTT TCT CG-3′; antisense 5′-GAC ACA TGG GGC ACC TTC TGA-3′) for the rat MMP-2 sequence and a 541 bp fragment using specific primers (sense 5′-CGG AGC ACG GGG ACG GGT ATC 3′; antisense 5′-AAG ACG AAG GGG AAG ACG CAC ATC 3′) for the rat MMP-9 sequence. In parallel, amplification of a 308 bp fragment of rat 18S rRNA (sense 5′-GCCTAGATACCGCAGCTAGGA-3′; antisense 5′-TCATGGCCTCAGTTCCGAA-3′), a relatively invariant internal reference gene, was performed. The primer sets, already validated in other studies (Latronico et al. 2013; Liuzzi et al. 2004; Gramegna et al. 2011) specifically recognize only the genes of interest as indicated by amplification of a single band of the expected size of the PCR products. The PCR amplification was carried out for 25 cycles each one consisting of denaturation at 94 °C, annealing at 59 °C and extension at 72 °C. After amplification, the products were analyzed in 1.5% agarose gels. After the densitometric analysis of gels, amplification of the target genes was normalized to 18S rRNA expression, and results were expressed as percentage of inhibition in comparison to positive control as reported for gelatinase quantification.

### Detection of ERK 1/2 phosphorylation by western blot analysis

ERK 1/2 were detected by immunoblot analysis as reported in Latronico et al. (2018). Briefly, confluent primary astrocytes plated in 6-well plates, made quiescent in serum-free medium for 24 h, were pretreated for 1 h with AITC (400 μM), PEITC (10 μM) or SFN (25 μM) in phenol red–free DMEM. After stimulation for 2 h with 10 μg mL^−1^ of LPS, cells were lysed with 20 mM Tris–HCl, 150 mM NaCl, 2.5 mM Na-pyrophosphate, 1 mM β-glycerophosphate 1% Triton X-100, 1 mM phenylmethylsulfonyl fluoride, 20 μg/mL aprotinin, 1 mM EGTA, 1 mM Na-fluoride, 1 mM Na_3_VO_4_, pH 7.5. Sixty μg of total proteins of each sample were resolved by 10% SDS–polyacrylamide gel electrophoresis and proteins were then immunoblotted onto polyvinylidene difluoride membranes. After blocking overnight at 4 °C with 0.05% Tween 20, 1% milk, 1% bovine serum albumin in 150 mM NaCl, 20 mM Tris–HCl (pH 7.5), membranes were probed overnight at 4 °C with a monoclonal anti-p-ERK 1/2 antibody (1:500). Then membranes were washed three times with 0.05% Tween 20 in Tris-buffered saline, probed with anti-mouse-horseradish peroxidase secondary antibody (1:20,000) for 2-h at 24 °C and detected with enhanced chemiluminescence. To assess the total amount of ERK, membranes were stripped and incubated with an antibody specific for non-phosphorylated ERK 1/2 (1:500). The band intensities were quantified by densitometric scanning of blots and levels of phosphorylation of ERK 1/2 were normalized to non-phosphorylated ERK 1/2 and expressed as percentage in comparison with positive control (astrocytes treated with LPS), according to the following equation:

% pERK 1/2 = (OD_sample_/OD_positive control_) × 100.

### Statistical analysis

Data analysis was performed by using the GraphPad Prism 5.0 (GraphPad Software Inc., San Diego, CA, USA). The data were expressed as mean values ± SD. Statistical analysis was carried out using one-way analysis of variance (ANOVA) followed by the Dunnett’s Multiple Comparison post hoc test.

## Results

### Effect of isothiocyanate compounds on astrocyte viability

**Fig. 1:**
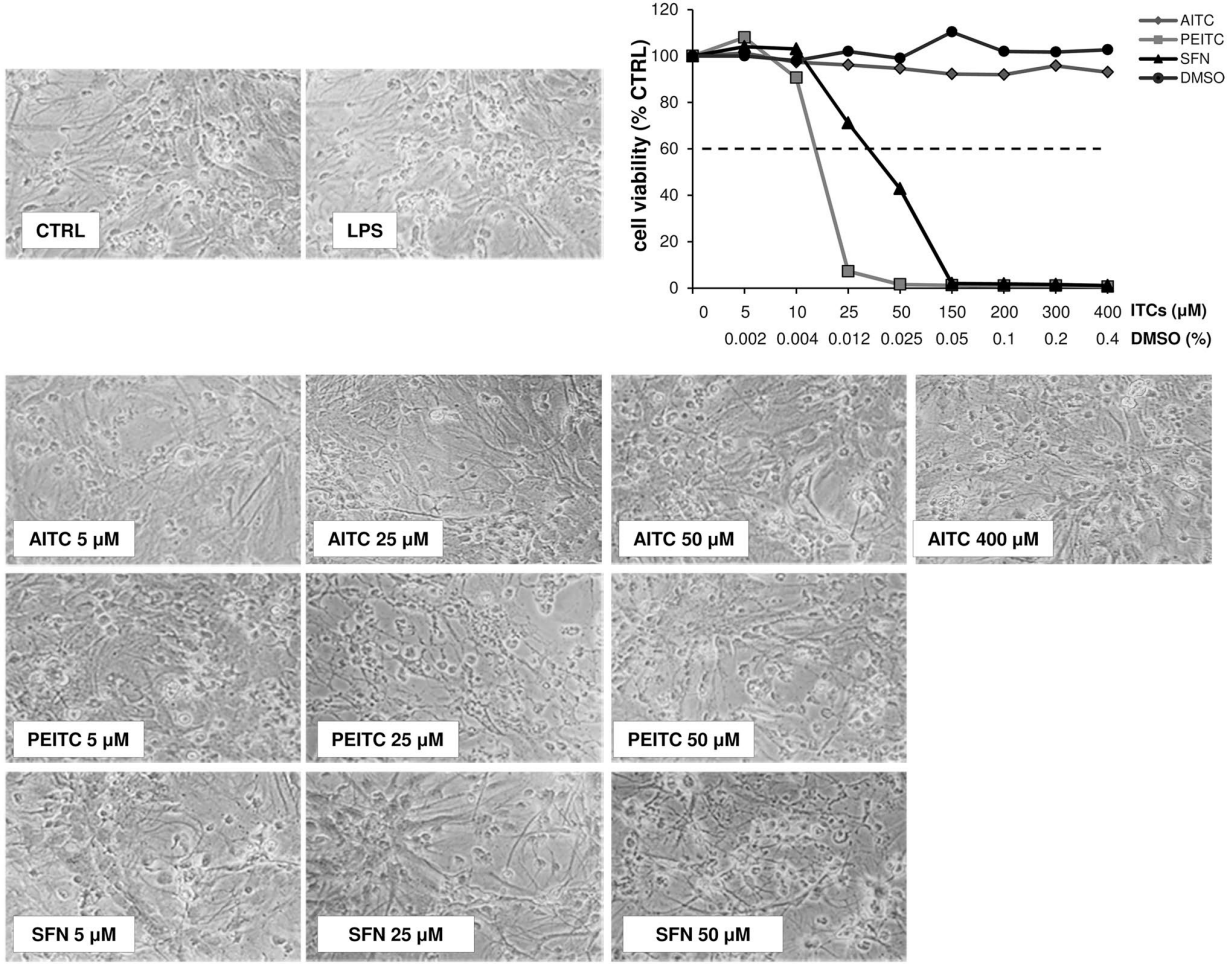
Cell viability of astrocytes treated with isothiocyanate compounds. Confluent primary astrocytes were treated for 20 h with Allyl isothiocyanate (AITC), 2-Phenethyl isothiocyanate (PEITC) or 2-Sulforaphane (SFN) at the indicated concentrations then subjected to the MTT assay. In addition, since ITCs in the stock solutions were dissolved in dimethyl sulfoxide (DMSO), a dose–response curve to DMSO, diluted in culture medium at the same solvent concentration (%) present in the ITC samples was run in each experiment to obtain a reliable estimation of ITC toxicity. The control (CTRL) was represented from untreated astrocytes in serum-free DMEM. The graphs represent the dose–response curves of cell viability, expressed as percentage of cell survival in comparison with control. The doses of compounds that determined a cell viability above 60% were chosen as the maximum non-toxic concentrations. Values are mean ± SD of *n* = 3 experiments performed on different cell populations. Micrographs show representative results of cell morphology observed under phase-contrast microscope (50X magnification) after treatment with the different compounds

Preliminary experiments were performed to evaluate the cytotoxicity of AITC, PEITC or SFN. To this purpose, purified astrocytes were treated for 20 h with the ITC compounds at concentrations ranging between 5 and 400 µM or with DMSO diluted in culture medium as described in the Method Section. As shown in Fig. 1, the DMSO was not toxic at all used concentrations that match to those of the tested ITC solutions. Among the investigated ITCs, AITC was the less toxic for astrocytes. In particular, AITC was not toxic for cells up to 400 µM while PEITC and SFN were toxic at concentration above 10 and 25 µM, respectively. The microscopic observation confirmed the results of the MTT assay showing that at the toxic concentrations of PEITC and SFN, astrocytes were reduced in number and those that remained showed cytotoxic changes in their cytoplasm.

### Protective effect of ITC compounds on ROS production in hydrogen peroxide-treated astrocytes

**Fig. 2:**
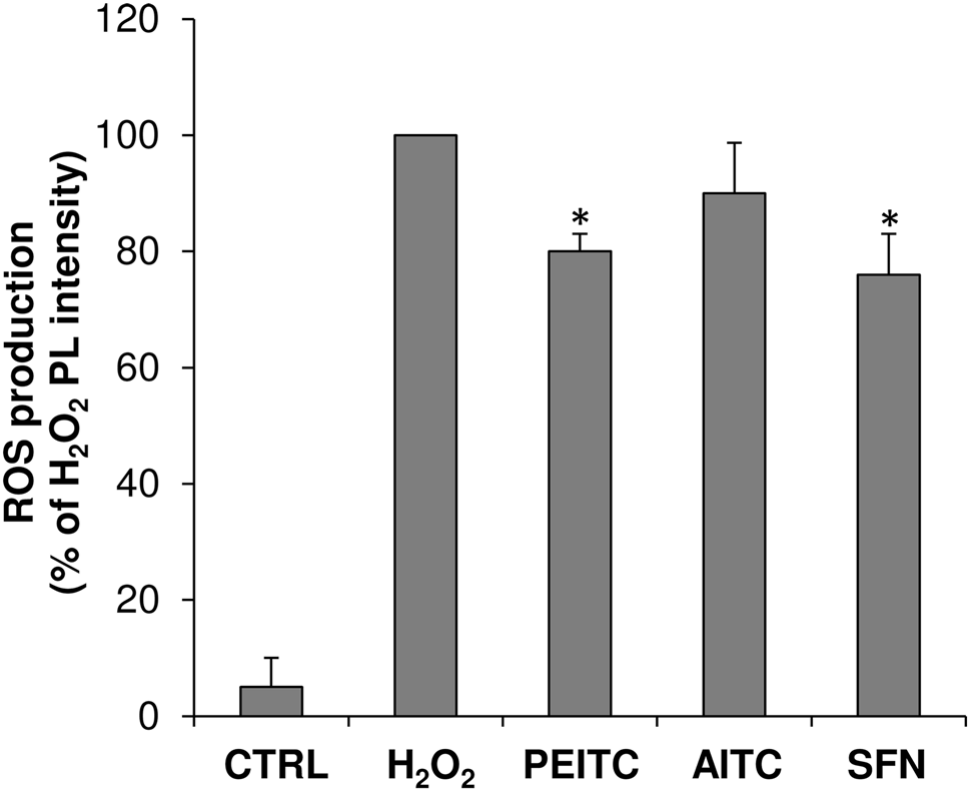
Production of reactive oxygen species (ROS) in astrocytes treated with ITCs. The presence of ROS was assayed measuring the changes of the fluorescent signal of 2′,7′-dichlorofluorescein (DCFA) as reported in the Materials and Methods section. Astrocytes, seeded in 6 well plates, were pre-treated for 30 min with DCFA then treated for 1 h with 10 μM PEITC, 400 μM AITC or 25 μM SFN in presence of H_2_O_2_ at final concentration of 100 μM. Astrocytes treated with DCFA alone (CTRL) or with 100 μM H_2_O_2_ represented negative and positive control, respectively. The fluorescent signal detected in cell lysates was measured by a fluorometer at 525 nm under excitation at 485 nm. The ROS production was expressed as percentage (%) of photoluminescence (PL) intensity in comparison to positive control. Values are mean ± SD of *n* = 3 experiments performed on different cell populations. Statistically significant decrease in comparison with H_2_O_2_ is indicated by asterisks (one-way ANOVA followed by Dunnet’s post hoc test; *p < 0.05)

To evaluate the protective effect of ITCs towards oxidative stress, astrocytes were pre-treated with PEITC, AITC and SFN at the maximum non-toxic concentration, then stimulated with H_2_O_2_. The intracellular ROS production, induced by H_2_O_2_, were assayed by DCFH-DA. As shown in Fig. 2, the exposure to H_2_O_2_ enhanced fluorescent signal in astrocytes in comparison to control (CTRL). As evidenced by the decrease of DCF fluorescence intensity, the pre-treatment of astrocytes with ITC compounds counteracted ROS generation induced by H_2_O_2_. Among the ITCs tested, only PEITC and SFN were able to significantly reduced ROS production of about 20 and 24%, respectively, in comparison to positive control (H_2_O_2_).

### The isothiocyanate compounds inhibit MMP-2 and MMP-9 levels in LPS-activated astrocytes

**Fig. 3:**
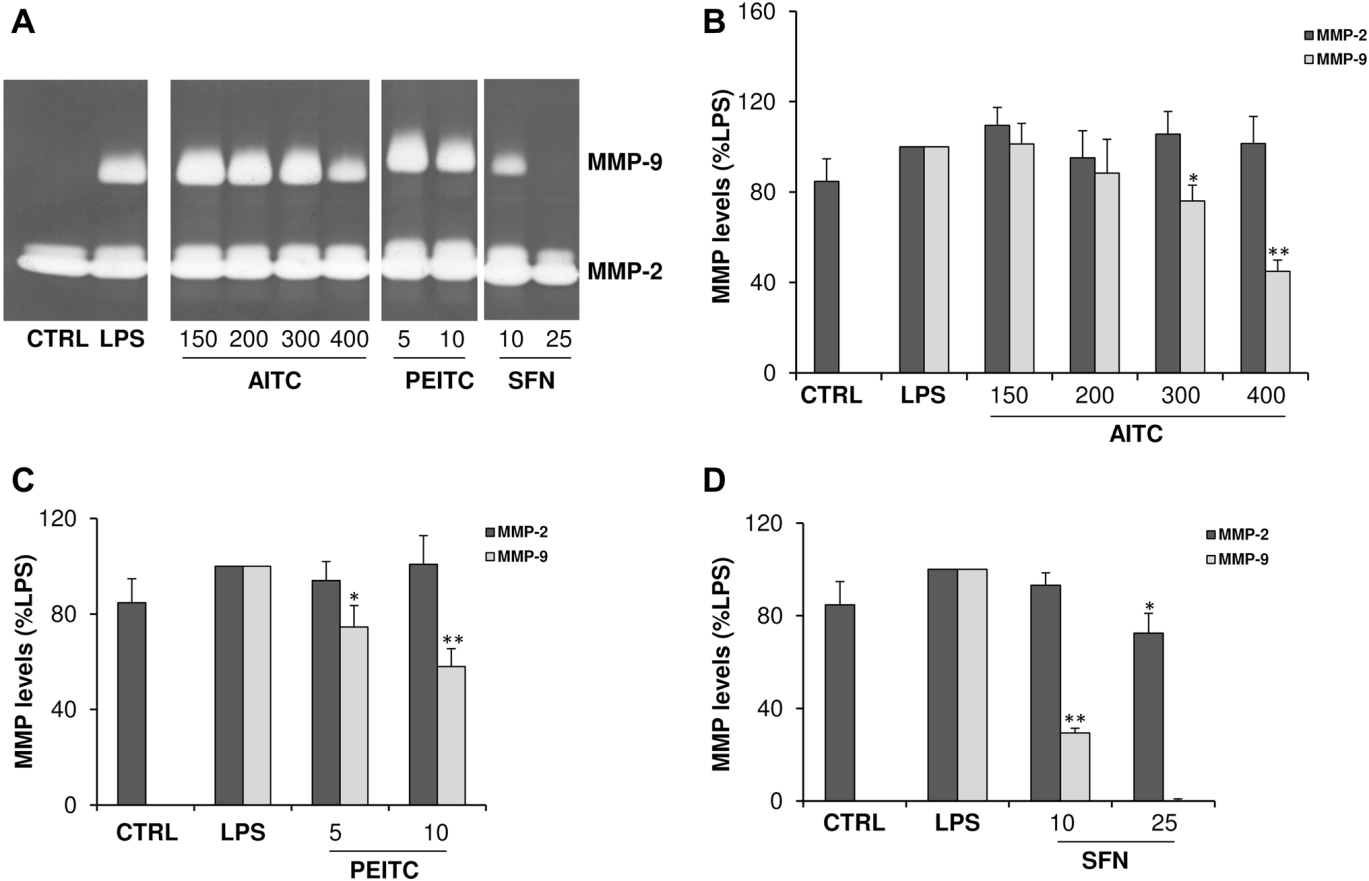
Effect of isothiocyanate compounds on MMP-2 and MMP-9 levels in LPS-activated astrocytes. In **a** is reported a representative zymographic gel of the analysis of culture supernatants from astrocytes activated with LPS (10 μg mL^-1^) and simultaneously treated for 20 h with AITC, PEITC or SFN at the indicated concentrations (μM). Negative and positive controls were obtained from unstimulated and untreated astrocytes in serum-free medium (CTRL) and LPS-activated cells (LPS), respectively. Histograms **b**–**d** represent results (mean ± SD) of *n* = 3 experiments performed on different cell populations, expressed as percentage of MMP inhibition in comparison with LPS, calculated after scanning densitometry and computerized analysis of gels. Statistically significant decrease in comparison with LPS is indicated by asterisks (one-way ANOVA followed by Dunnet’s post hoc test; *p < 0.05, **p < 0.01)

The effect of ITC compounds on MMP-2 and MMP-9 levels was evaluated on astrocytes activated with LPS, which is known to induce the expression of gelatinases (Gottschall and Deb 1996), and simultaneously treated with AITC, PEITC or SFN in the range of concentrations that were not toxic for cells. As shown in Fig. 3a, two clear bands of digestion of 72 and 92 kDa were present on the gel corresponding, respectively, to MMP-2 and MMP-9. The activation of astrocytes with LPS increased levels of MMP-2 and induced the synthesis of MMP-9. Treatment with AITC (Fig. 3b), PEITC (Fig. 3c), or SFN (Fig. 3d) determined a dose-dependent reduction of MMP-9 levels, which varied depending on the compound used. In particular, among the ITC used, SFN was the most effective in inhibiting MMP-9 since it determined 70% inhibition at the dose of 10 μM and 100% inhibition at the dose 25 μM. In addition, only SFN at the maximum non-toxic concentration was able to significantly inhibit MMP-2 (Fig. 2d).

### The isothiocyanate compounds inhibit MMP-2 and MMP-9 gene expression in LPS-activated astrocytes

**Fig. 4:**
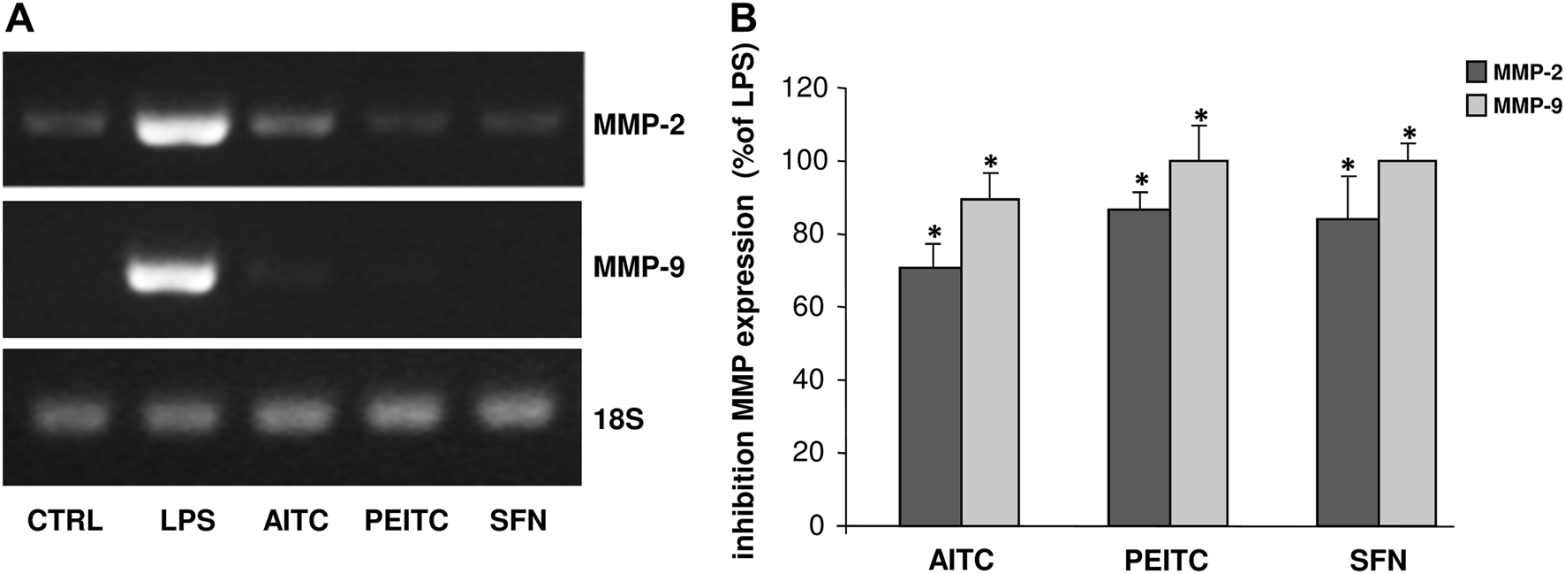
Effect of ITC compounds on MMP-2 and MMP-9 expression in LPS-activated astrocytes. Astrocytes, seeded in 6 well plates, were activated with LPS (10 µg mL^-1^) and simultaneously treated for 20 h with AITC (400 μM), PEITC (10 μM) or SFN (25 μM), then subjected to RT-PCR. Representative agarose gels of MMP-2 and MMP-9 expression are reported in (**a**). Histograms in (**b**) represent results (mean ± SD) of *n* = 3 experiments performed on different cell populations, expressed as percentage of MMP inhibition in comparison with positive control (LPS), calculated after scanning densitometry and computerized analysis of gels. Statistically significant decrease of MMP-2 and MMP-9 expression in comparison with LPS is indicated by asterisks (one-way ANOVA followed by Dunnet’s post hoc test; *p < 0.01)

To determine whether the studied ITC compounds were able to inhibit MMP-2 and MMP-9 expression, RT-PCR was performed on LPS-activated astrocytes treated with the maximum non-toxic concentration of ITCs. As shown in the representative gels in Fig. 4a the treatment of LPS-activated astrocytes with the individual ITCs was able to counteract the increased expression of MMP-2 and MMP-9 mRNA. As shown in Fig. 4b, the quantitative analysis of results, evidenced that, at the maximum non-toxic concentration, the ITC compounds, determined a statistically significant inhibition of MMP-2 and MMP-9 expression in comparison to positive control (LPS). In particular, PEITC and SFN exert the same percentage of inhibition on MMP-2 (85%) and MMP-9 (100%) whereas AITC was less effective than PEITC and SFN in inhibiting MMP-2 (65%) and MMP-9 (90%) expression.

### ERK 1/2 is involved in the inhibition of MMP expression by ITC

Since ERK 1/2 is the main signaling transduction pathway involved in the regulation of MMP-2 and MMP-9 expression in LPS-activated astrocytes (Lee et al. 2003), the effect of ITC compounds on the activation of extracellular-regulated protein kinases (ERK) 1/2 was tested.

**Fig. 5:**
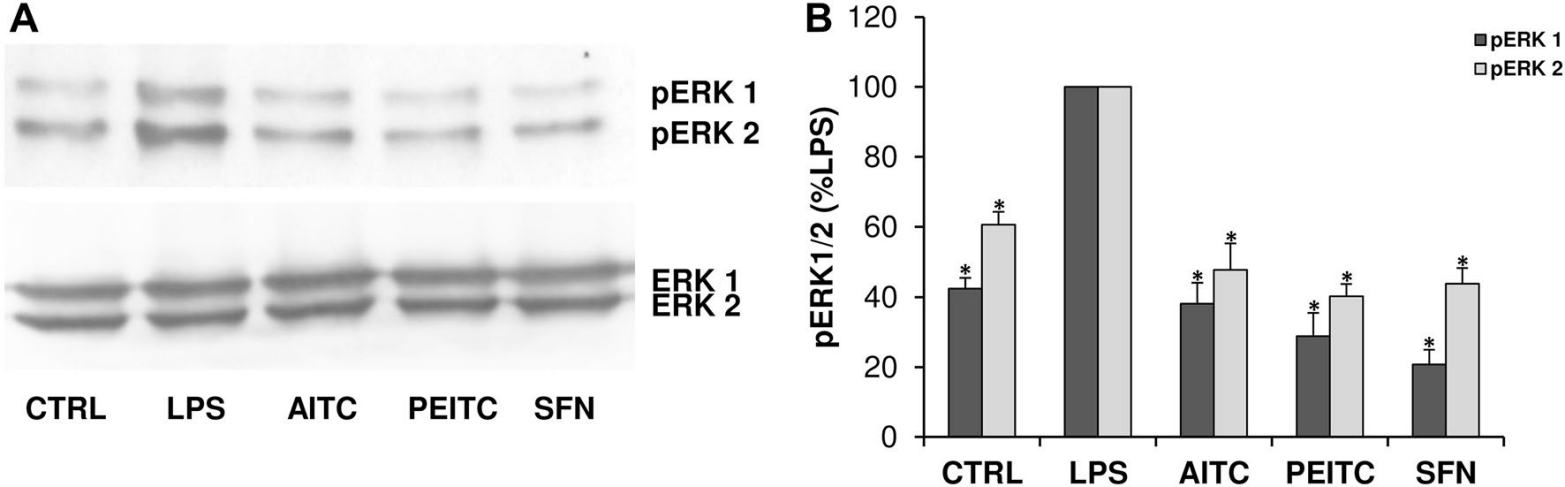
Extracellular signal-regulated kinase 1/2 (ERK 1/2) is involved in the inhibition of MMP expression by ITC compounds. **a** Representative autoradiographic films of western blotting analysis of lysates from astrocytes pre-treated for 1 h with AITC (400 μM), PEITC (10 μM) or SFN (25 μM), and then activated for 2 h with LPS (10 μg mL^-1^). Untreated and unstimulated cells represent negative control (CTRL). Histogram in (**b**) represents pERK1/2 levels obtained after densitometric scanning of autoradiographic films and normalization to non-phosphorylated ERK 1/2. Data represent means ± SD of *n* = 3 experiments performed on different cell populations. Asterisks represent values statistically different from positive control (LPS-activated astrocytes), which was set at 100% (one-way ANOVA followed by the Dunnett’s Multiple Comparison post hoc test; *p < 0.01)

As shown in Fig. 5 LPS induced a statistically significant increase in levels of phosphorylated ERK 1/2 (p-ERK) that was counteracted by pre-treatment with the ITC compounds.

## Discussion

It is widely believed that the main defence strategies against many diseases consist mainly in avoiding risk factors and adopting a correct lifestyle. There are numerous scientific evidences supporting the hypothesis that some natural metabolites, typically produced by various kinds of fruits and vegetables are able to modulate pathological phenomena such as cancer, neurological diseases and other multifactorial diseases. In this scenario, the isothiocyanates (ITCs) aroused great interest given their antifungal, antibacterial, anticarcinogenic, antimutagenic, antioxidant and antinflammatory biological properties (Dufour et al. 2015; Marzocco et al. 2015; Vig et al., 2009; Talalay and Fahey 2001). Isothiocyanates originate from the enzymatic degradation of glucosinolates (GSL) which are secondary metabolites present in 15 botanical families of the Capparales order and are very abundant in the Brassicaceae family (Fahey et al. 2001). GSL are hydrolyzed by myrosinase (β-thioglucoside glucohydrolas e; EC 3.2.3.147) in a variety of components such as ITCs, nitrites, thiocyanates, epithionitriles, SFNate, glucose and oxazolidines (Li and Kushad 2005). Although ITCs have been extensively studied in the chemotherapeutic field (Grundemann and Huber 2018), few investigations have been made about their therapeutic potential in neurological diseases despite it has been demonstrated the ability of SFN and AITC to cross the BBB and accumulate into the brain parenchyma (Clarke et al. 2011; Jazwa et al. 2011; Zhang 2010). In this paper we compared the effects of three ITCs, present in several commonly consumed cruciferous vegetables, on reactive oxygen species (ROS) production and matrix metalloproteinase (MMP) expression in an in vitro model of neuroinflammation represented by astrocytes activated with lipopolysaccharide (LPS) or treated with H_2_O_2_. Astrocytes, the most abundant glial cell type of the CNS, play a crucial role for the maintenance of brain homeostasis and are hallmark of different neurological diseases (Cekanaviciute and Buckwalter 2016; Colombo and Farina 2016). They are considered crucial regulators of the innate immune response and their response to harmful insults, such as oxidative stress, may exacerbate inflammatory reactions and tissue damage or promote tissue repair. It is well known that activated astrocytes produce neurotoxic factors such as MMPs which have a key role in promoting neuroinflammation and progressive neuronal loss in neurological diseases (Hsieh and Yang 2013). Therefore, the modulation of the events that occur following activation of astrocytes could mitigate the inflammatory response and neuronal damage.

Results of this study indicated that among the ITCs studied, AITC was the less toxic as it did not result in any reduction in cell viability at all concentrations used. By contrast, similarly to what has been observed by other authors on various glial cell lines, PEITC and SFN showed cellular toxicity at concentrations greater than 10 and 25 µM, respectively (Lee et al. 2015; Eren et al. 2018; Su et al. 2015).

In the current scientific literature there are many controversial data on the molecular mechanisms underlying the effects of ITCs on cell viability. In this respect, most of the studies have suggested that ITCs inhibit the cellular growth inducing apoptosis, cell cycle arrest, generation of ROS in cancer cells but not in normal cells (Fofaria et al. 2015; Qin et al. 2018; Sestili and Fimognari 2015).We found that SFN and PEITC, but not AITC, have a protective role against oxidative stress in astrocytes, indeed at the maximum non-toxic concentrations they were able to counteract ROS production induced by exposure to H_2_O_2_. The CNS is particularly vulnerable to oxidative stress due to its high oxygen consumption, weakly antioxidant systems and the high content of biomacromolecules susceptible to oxidation. The generation of ROS and oxidative damage is believed to be involved in the pathogenesis of neurological disorders such as Alzheimer’s disease (AD), Parkinson’s disease (PD), Amyotrophic Lateral Sclerosis (ALS) and Multiple Sclerosis (MS) (Lee et al. 2015). Therefore, although in our in vitro experiments SFN and PEITC counteracted ROS production to a moderate extent, we might suppose that in vivo a prolonged administration of ITCs can lead to a more significant reduction in ROS that, over time, can result meaningful for the prevention and modulation of brain diseases. ROS act as a critical signaling molecules to trigger the cascade of inflammatory response into CNS. In this respect, it is well known that ROS regulate the expression of many genes involved in neuroinflammation through the activation of pro-inflammatory transcription factors such as nuclear factor-κB (NF-κB) (Lee et al. 2015; Chiurchiù et al. 2016; Martorana et al. 2019). In this scenario an important role is played by MMPs whose expression is up-regulated by ROS through the activation of NF-κB (Yan and Boyd 2007).

Since MMP dysregulation contributes to the pathogenesis of numerous neuroinflammatory and neurodegenerative diseases, their inhibition represents an important therapeutic strategy. For this reason, growing interest has been focused in the last decade on the potential of drugs and natural compounds to inhibit MMPs. Numerous in vitro studies have evidenced that anti-inflammatory and antioxidant action of several drugs and bioactive compounds present in vegetables and marine organisms are associated with their abilities to inhibit the expression of MMPs (Latronico et al. 2016, 2018; Di Bari et al. 2015). In this study we demonstrated that among the tested ITCs, SFN and PEITC were the most effective in inhibiting MMP-2 and MMP-9 levels and expression in LPS-activated astrocytes. The inhibition of MMPs by ITCs has been reported in other in vivo and in vitro studies. In particular, Li et al. (2013) demonstrated that SFN is able to mitigate the BBB damage by inhibiting MMP-9 expression in an in vivo model of experimental autoimmune encephalomyelitis. Other authors reported that SFN was able to attenuate MMP-9 expression following spinal cord injury in mice (Mao et al. 2010a). In addition, in vitro tudies demonstrated that the suppression by ITCs of cell migration and invasion in C6 glioma cells was associated to inhibition of MMP-9 expression mediated by NF-κB (Lee et al. 2015).

The molecular mechanisms by which ITCs inhibit MMPs is unclear, and there are no studies investigating the antioxidant and anti-inflammatory properties of ITCs on primary astrocyte cultures. Several studies support the hypothesis that the inhibitory effect of ITCs is a consequence of their ability to block NF-κB by Nrf2 signaling pathway. In this respect, an in vivo study evidences that Nrf2 knockout mice were more susceptible to NF-κB-mediated inflammatory response of spinal cord and to the increased expression of NFκB-dependent genes including MMP-9 (Mao et al. 2010b). Although NF-κB and Nrf2 pathways could interplay we demonstrated that the regulation of NF-κB occurs also through the modulation of molecular mechanisms that are independent by Nrf2. Indeed, we found that AITC, PEITC and SFN inhibited the activation of ERK1/2 which play a pivotal role in the cascade of signal transduction events that ultimately regulate MMP gene transcription.

However, the experimental evidence that SFN at the maximum non-toxic concentration completely inhibited both MMP-9 expression and levels whereas AITC and PEITC inhibited to a maximum extend MMP-9 expression but only dropped MMP-9 levels to around 50%, might suggest that these compounds modulate MMP-9 transcription with different mechanisms of action. In this respect, it has been reported that SFN, in addition to the inhibition of LPS-induced activation of NF-κB, interferes with the binding of LPS to TLR4 receptor (Koo et al 2013). The inhibitory effect of SFN on TLR4 could prevent the activation of astrocytes by LPS and this may result in the upstream inhibition of MMP-9 expression with a consequent lack of MMP-9 production.

## Conclusions

In summary, our data indicate that treatment of astrocytes with ITCs reduces ROS formation and inhibits the release of MMP-2 and MMP-9 providing new insights into the antioxidant and anti-inflammatory properties of ITCs on glial cells. These results suggest that ITCs could be promising nutraceutical agents for the development of neuro-nutrition interventions, based on the use of healthy foods, for the prevention and complementary treatment of neurological diseases.
